# In-Lab Upfront Use of Tirofiban May Reduce the Occurrence of No-Reflow During Primary Percutaneous Coronary Intervention. A Pilot Randomized Study

**DOI:** 10.5935/abc.20160149

**Published:** 2016-11

**Authors:** Igor Matos Lago, Gustavo Caires Novaes, André Vannucchi Badran, Rafael Brolio Pavão, Ricardo Barbosa, Geraldo Luiz de Figueiredo, Moysés de Oliveira Lima, Jorge Luiz Haddad, André Schmidt, José Antônio Marin

**Affiliations:** 1 Divisão de Cardiologia, Departamento de Clínica Médica, Hospital das Clínicas da Faculdade de Medicina de Ribeirão Preto - Universidade de São Paulo, São Paulo, SP - Brazil; 2 Hospital São Joaquim, Franca, SP - Brazil

**Keywords:** Coronary Artery Disease, Myocardial Infarction, Percutaneous Coronary Intervention, Platelet Glycoprotein GPIIb-IIIa Complex, Angioplasty

## Abstract

**Background:**

Despite successful opening of culprit coronary artery, myocardial reperfusion does not always follows primary percutaneous coronary intervention (PPCI). Glycoprotein IIb/IIIa inhibitors are used in the treatment of no-reflow (NR), but their role to prevent it is unproven.

**Objective:**

To evaluate the effect of in-lab administration of tirofiban on the incidence of NR in ST-elevation myocardial infarction (STEMI) treated with PPCI.

**Methods:**

STEMI patients treated with PPCI were randomized (24 tirofiban and 34 placebo) in this double-blinded study to assess the impact of intravenous tirofiban on the incidence of NR after PPCI according to angiographic and electrocardiographic methods. End-points of the study were: TIMI-epicardial flow grade; myocardial blush grade (MBG); resolution of ST-elevation < 70% (RST < 70%) at 90min and 24h after PPCI.

**Results:**

Baseline anthropometric, clinical and angiographic characteristics were balanced between the groups. The occurrence of TIMI flow < 3 was not significantly different between the tirofiban (25%) and placebo (35.3%) groups. MBG ≤ 2 did not occur in the tirofiban group, and was seen in 11.7% of patients in the placebo group (p=0.13). RST < 70% occurred in 41.6% x 55.8% (p=0.42) at 90min and in 29% x 55.9% (p=0.06) at 24h in tirofiban and placebo groups, respectively. Severe NR (RST ≤ 30%) was detected in 0% x 26.5% (p=0.01) at 90 min, and in 4.2% x 23.5% (p=0.06) at 24h in tirofiban and placebo groups, respectively.

**Conclusion:**

This pilot study showed a trend toward reduction of NR associated with in-lab upfront use of tirofiban in STEMI patients treated with PPCI and paves the way for a full-scale study testing this hypothesis.

## Introduction

After sudden death, acute ST-segment elevation myocardial infarction (STEMI) is the second most severe clinical presentation of coronary artery disease (CAD) in the US and Europe.^1,2^ For more than one decade, primary percutaneous coronary intervention (PPCI) has been considered the most appropriate treatment to restore myocardial blood flow and, consequently, favorably impact survival^3^ . Despite epicardial coronary flow being established in the culprit artery, a substantial proportion of patients do not achieve adequate myocardial reperfusion, known as the no-reflow (NR) phenomenon.^4^ The NR phenomenon has a negative influence on PPCI^5^ due to its association with a greater myocardial necrosis area and more intense irreversible left ventricular systolic dysfunction, which are independent predictors of mortality.^6^ Various therapeutic measures including pharmacologic approaches and mechanical devices have been used to manage the two principal pathophysiological mechanisms leading to NR, i.e. microvascular spasm, and clot and distal embolism of plaque debris.^7^ However, the role of each of these therapeutic resources, including the use of IIb/IIIa glycoprotein inhibitors (GPI), is still inconclusive, especially for the prevention of NR. Hence, with the current therapeutic management of patients with STEMI undergoing PPCI, that includes dual antiplatelet therapy and antithrombin agents, an investigation about the systematic upfront in-lab use of a GPI to lower the occurrence of NR is appropriate, especially when bare-metal stents are implanted, as in most developing countries. This was the scope of this pilot randomized study, which compared the incidence of NR in STEMI patients treated with PPCI and tirofiban or placebo.

## Methods

From August 2011 through January 2014, 64 patients with STEMI referred for PPCI by the emergency health care system were enrolled at two tertiary hospitals, located in Ribeirão Preto and in Franca, state of São Paulo, Brazil. Patients or their responsible relatives signed informed consent forms, approved by the Ethics Committee of Ribeirão Preto General Hospital, University of São Paulo Medical School , responsible for the two hospital centers participating in the investigation (Process 2495/2010).

Using a centralized, unrestricted (simple) randomization system, 58 patients were randomized to receive intravenous infusions of tirofiban (n=24) or placebo (n=34 ) in a double blind manner. The inclusion criteria consisted of: age ≥18 years, typical thoracic pain ≥ 20 minutes and < 12 hours duration, ST segment elevation ≥ 1mm in two contiguous leads or presumed new left bundle-branch block. Six of the referred patients were excluded, based on the following criteria: cardiogenic shock, prior myocardial infarction in the same ventricular wall as the current coronary event, known bleeding diathesis, coma, severe hepatic dysfunction or severe renal insufficiency (Creatinine > 3.0 mg/dL), contraindications for acetylsalicylic acid (ASA) , clopidogrel or heparin, life expectancy <1 year, previous major surgery < 3 months, stroke < 30 days, previously known intracranial aneurism or arteriovenous malformation, severe trauma < 6 weeks, use of oral anticoagulant; inability to give written informed consent.

Immediately after randomization of patients , the infusion solutions were prepared by a registered nurse, who was the only person aware of which treatment was administered to each person . Placebo or tirofiban (25mcg/Kg) were administered intravenously in bolus of 10 ml over 3min as soon as the patients entered in the catheterization laboratory. In both arms of the trial, equivalent volumes were then infused for 12 hour s, so that tirofiban was administered at the dose of 0.15mcg/Kg/min. Unfractionated heparin boluses were administered in a dose of 70U/Kg for the patients in the tirofiban group and of 100U/Kg in the placebo group, by the same nurse who prepared the solutions . All other clinical and laboratory procedures related to the PPCI after the patient left the cath lab, regarding medications, use of thrombus aspiration catheters, and laboratory tests were left at the discretion of the interventional physician. The therapeutic procedure was limited to the infarct related culprit artery. NR was diagnosed through angiographic epicardial flow and myocardial blush grade (MBG) criteria, by the attending physician during PPCI, to assess the requirement for other medications, including intracoronary nitroglycerin, adenosine or verapamil. After the end of PPCI, a blinded reevaluation of the angiographic grading of epicardial flow and myocardial blush was performed by a consensus of two experienced interventionists. They also blindly evaluated the occurrence of NR by analysis of ST segment elevation resolution on the electrocardiogram at 90 minutes and 24 hours post-PPCI. In case of discordance, a third opinion was called to achieve a consensus. Time intervals assessed in the study included time from onset of chest pain to arrival at the emergency room , time from initial pain to arrival at the cath lab, and duration of the diagnostic and therapeutic procedures. Clinical complications and duration of hospitalization were also recorded.

Sample size was calculated assuming a NR incidence of 70% in the placebo group based on a ST segment resolution > 70% (no resolution) at 90 minutes after the PPCI . The study hypothesis was that, using this criterion, NR incidence would be reduced by 50% in the tirofiban group, *i.e.*, an expected incidence of about 35%, with 80% power and two-tailed α = 0.05. The calculation resulted in a total of 56 patients, with 28 in each group.^8^ Statistical analysis was performed using the programs STATA.^9^ The normal distribution of each variable analyzed was verified by Shapiro-Wilk test; the quantitative variables with normal distribution were described as mean (M) and standard deviation (± SD), and the categorical variables were described as absolute values and as frequency (n) and percentage (%). Continuous variables with and without normal distribution were analyzed by parametrical Student's t-test and Mann-Whitney test, respectively. Chi-square and Fisher exact tests were used for categorical variables. Multivariable analysis by logistic regression was made to identify independent predictors of post-PPCI ST resolution with the following dichotomous variables: tirofiban/placebo use; PPCI initial time, time from onset of chest pain; post-PPCI Thrombolysis In Myocardial Infarction (TIMI) flow grade ; coronary nitrate and adenosine administration; use of catheters for the mechanic aspiration of thrombus; stent post-dilation during PPCI. The significance level of 0.05 was set for all comparisons.

## Results

Of the 64 patients enrolled, 6 had to be excluded after randomization - 3 for protocol violations, 1 due to absence of culprit lesion and 1 because of ensuing cardiogenic shock. Of the remaining 58 patients, 34 patients were allocated to the placebo group and 24 patients to the tirofiban group. The main baseline clinical characteristics including previous medications were similar between groups (Table 1). With regard to the 3 patients with previous coronary events all were in the tirofiban group and had previous P PCI (p=0.06). Cocaine use was identified in 1 patient of the placebo group (p=1.00). The majority of patients in both groups reached the cath lab within 6 hours of the onset of symptoms (83.3% in the tirofiban group and 77.6% in the placebo group), and most of them were classified as Killip-Kimball class I or II.

**Table 1:** Baseline characteristics

	Tirofiban (%) ^¶^	Placebo (%) ^Ω^	p
Patients	24 (41.4%)	34 (58.6%)	
Age	59.5 (±10.5)	58.3 (±11.9)	(=0.70) ^Ţ^
Male	19 (79.2%)	30 (88.2%)	(=0.46) **^ƒ^**
Caucasian	22 (91.6%)	27 (79.4%)	(=0.28) ^ƒ^
BMI ≥ 30	5 (20.8 %)	7 (20.6%)	(=1.00) ^ƒ^
Hypertension	16 (66.6%)	23 (67.6%)	(=0.84) ^χ^
Smokers	13 (54.2%)	16 (47.1%)	(=0.79) ^χ^
Dyslipidemia	10 (41.6%)	11 (32.4%)	(=0.58) ^χ^
CAD Family History	5 (20.8%)	12 (35.3%)	(=0.26) ^ƒ^
Diabetes	3 (12.5%)	7 (20.6%)	(=0.49) ^ƒ^
ACEI/ARB	10 (41.6%)	15 (44.1%)	(=1.00) ^ƒ^
Oral antidiabetic	3 (12.5%)	8 (23.5%)	(=0.33) ^ƒ^
Statin	6 (25%)	3 (8.8%)	(=0.14) ^ƒ^
ASA	2 (8.3%)	4 (11.7%)	(=1.00) ^ƒ^
Diuretics	1 (4.2%)	5 (14.7%)	(=0.38) ^ƒ^

BMI: body mass index; CAD: coronary artery disease; ACEI: angiotensin converting enzyme inhibitor; ARB: angiotensin receptor blocker; ASA: acetylsalicylic acid; p: p-value;

ƒ p-value calculated by Fisher Exact Test;

Ţ p-value calculated by “t” Student Test (95% IC 4.86-7.26);

Χ p-value calculated by Chi-square Test;

¶ tirofiban percentage group;

Ω placebo percentage group

Table 2 shows technical aspects of the diagnostic cardiac catheterization procedure that was performed using right radial arterial approach in 23 (95.8%) of tirofiban patients and in 31 (91.2%) placebo patients (p=1.00). In 57 patients (98.3%) a 6F sheath was inserted. Per protocol, all patients in both groups were treated with ASA 300mg, and clopidogrel 300mg before the cardiac catheterization was initiated. Left contrast ventriculography was done at the end of the procedure in 44 (75.6%) patients, 19 (79.2%) from the tirofiban group and 25 (73.5%) from the placebo group. Moderate impairment of global systolic left ventricular (LV) function occurred 15 (60%) patients of the placebo group, opposed to 5 (26.3%) of the tirofiban group (p=0.03). No other angiographic differences were seen when comparing both groups (Table 2), including the duration of the diagnostic procedure with an average of 16.7 (±11.2) minutes in the tirofiban group and of 13.7 (±9.8) minutes in the placebo group (p=0.11).

**Table 2:** Characteristics of diagnostic catheterization

LV/EF	(19 Tirofiban) ^¶^ (%) υ	(25 Placebo)^Ω^ (%) υ	p
< 30%	4 (21.1%)	2 (8%)	(=0.34) ^ƒ^
31-40%	5 (26.3%)	15 (60%)	(=0.03) ^ƒ^
41-50%	6 (31.5%)	6 (24%)	(=0.73) ^ƒ^
No Ventriculography	5 (26.3%)	9 (36%)	(=0.53) ^ƒ^
CAD pattern and grade of luminal obstruction			
UNIARTERIAL	14 (58.3%)	21 (61.7%)	(=1.00) ^ƒ^
BIARTERIAL	7 (29.2%)	8 (23.5%)	(=0.76) ^ƒ^
TRIARTERIAL	3 (12.5%)	5 (14.7%)	(=1.00) ^ƒ^
100 %	19 (79.2%)	29 (85.3%)	(=0.73) ^ƒ^
71 - 99 %	5 (20.8%)	4 (11.7%)	(=0.46) ^ƒ^
51 - 70 %	0 (0%)	1 (2.9%)	(=1.00) ^ƒ^

LV: left ventriculography; EF: ejection fraction; CAD: coronary artery disease; p: p-value;

ƒ p value calculated by Fisher Exact Test;

υ only for patients that were submitted to LV;

¶ tirofiban group percentage;

Ω p lacebo group percentage.

The main characteristics of the therapeutic procedure are shown on table 3. All patients were instrumented with the same arterial approach and sheath diameters used for the diagnostic procedure. Bare metal stents were exclusively used in all procedures, with a similar rate in both groups, 1.37 stent/patient in the tirofiban group and 1.29 stent/patient in the placebo group (p=0.75). PPCI with balloon was performed in only one patient allocated to the tirofiban group. A trend for more post-stent dilation was observed in the placebo group (p=0.06). The time from patients' arrival at the ca th lab to the start of intravenous administration of tirofiban or placebo was similar in both groups, 6.26 (±3.1) minutes in the tirofiban group and 8.52 (±6.4) minutes in the placebo group. Clinical complications occurred in similar proportions of patients in both groups: systolic arterial pressure < 90mmHg in 5 (20.8%) tirofiban patients and in 5 (14.7%) placebo patients (p=0.73); heart rate > 100bpm in 4 (16.6%) tirofiban patients and in 2 (5.8%) placebo patients (p=0.22); bradyarrhythmia in 4 (16.6%) tirofiban patients and in 1 (2.9%) placebo patient (p=0.15). One (1.7%) tirofiban patient had a successfully resuscitated cardiac arrest (p=0.41); severe hypotension or shock was present in 3 (12.5%) tirofiban patients and in 2 (5.8%) placebo patients (p=0.64); small hematoma at the vascular access site , according to TIMI classification, was present in 4 (16.6%) tirofiban patients and in 3 (8.8%) placebo patients (p=0.43); hemorrhagic stroke occurred in 1 patient of the placebo group 3 days after PPCI (p=1.00). The duration of the therapeutic procedure was similar in both groups, 31.8 (± 20.2) minutes in tirofiban group and of 35.4 (± 22.7) minutes in placebo group (p=0.47). Thrombus manual aspiration during the therapeutic procedure and utilization of other medications were also similar in both groups (Table 3).

**Table 3:** Characteristics of therapheutic procedure

PPCI	Tirofiban(%) ^¶^	Placebo (%) ^Ω^	p
Direct Stent	7 (29.2%)	14 (41.2%)	(=0.41) ^ƒ^
Predilation	17 (70.8%)	20 (58.8%)	(=0.41) ^ƒ^
Postdilation	1 (4.2%)	8 (23.5%)	(=0.06) ^ƒ^
Maximal Stent Pressure	13.4±2.1 **υ**	13.8±2.4 **υ**	(=0.10) ^Σ^
≤ 12	16 (20.%) **υ**	10 (12.9%)**υ**	(=0.03) ^ƒ^
13 A 15	11 (37.9%) **υ**	22 (52.4%)**υ**	(=0.18) ^ƒ^
≥16	6 (7.8%) **υ**	12 (15.6%)**υ**	(=0.42) ^ƒ^
**Nr Therapy**			
Nitroglycerin	7 (29.2%)	13 (38.2%)	(=0.58) ^ƒ^
Adenosine	3 (12.5%)	10 (29.4%)	(=0.21) ^ƒ^
**Manual Aspiration of Thrombus**	4 (16.6%)	7 (20.6%)	(=1.00) ^ƒ^

PPCI: primary percutaneous coronary intervention; NR: no reflow; p: p value;

Σ p value calculated by Mann-Whitney Test;

ƒ p-value calculated by Fisher Exact Test;

υ numbers of sent values;

¶ tirofiban group percentage;

Ω placebo group percentage.

According to the angiographic criteria of epicardial TIMI flow grade < 3, the incidence of NR at the end of PPCI was not different between groups: 6 (25%) tirofiban patients and in 12 (35.3%) placebo patients (Table 4). By the angiographic criteria of MBG < 2 at the end of PPCI, NR did not occur in any patient of the tirofiban group and was detected in 4 patients of the placebo group (11.7%), but this difference was not statistically significant. By the ECG criteria (STR < 70%), NR occurred in 10 patients (41.6%) in the tirofiban group and 19 patients (55.8%) in the placebo group at 90 minutes after the PPCI (p=0.42). Also, 7 (29%) of tirofiban patients and 19 (55.8%) placebo patients met the criteria for NR at 24 hours (p=0.06). When the criteria of severe NR (STR < 30%) was used, no tirofiban patient and 9 (26.5%) placebo patients met it at 90 minutes (p=0.01) and 1 (4.2%) tirofiban patient and 8 (23.5%) placebo patients had severe NR at 24 hours (p=0.06).

**Table 4:** Assessment of reperfusion achieved with ppci

TIMI/MBG GRADES	Tirofiban (%) ^¶^	Placebo (%) ^Ω^	p
**TIMI PRE-PPCI**			0-2 x 3
0	22 (91.6%)	29 (85.3%)	(=1.00) ^ƒ^
1	0(0%)	1 (2.9%)	
2	2 (8.3%)	2(5.8%)	
3	0(0%)	2 (5.8%)	
**TIMI POST-PPCI**			0-2 x 3
0	0(0%)	0(0%)	(=0.40) ^ƒ^
1	0(0%)	1 (2.9%)	
2	6 (25%)	12 (35.3%)	
3	18 (75%)	21 (61.7%)	
**MBG PRE-PPCI**			0-1 x 2-3
0	22 (91.6%)	29 (85.3%)	(=1.00) ^ƒ^
1	0 (0%)	2 (5.8%)	
2	2 (8.3%)	0 (0%)	
3	0 (0%)	3 (8.8%)	
**MBG POST-PPCI**			0–1 x 2–3
0	0 (0%)	0 (0%)	(=0.13) ^ƒ^
1	0(0%)	4 (11.7%)	
2	11 (45.8%)	10 (29.4%)	
3	13 (54.2%)	20 (58.8%)	
ECG/STR	90 min	90 min	
No STR (<30%)	0(0%)	9(26.5%)	(=0.01) ^ƒ^
STR (71-100%)	14 (58.3%)	15 (44.1%)	(=0.42) ^ƒ^
ECG/STR	24h	24h	
NO STR (<30%)	1(4.2%)	8 (23.5%)	(=0.06) ^ƒ^
STR (71-100%)	17 (71%)	15 (44.1%)	(=0.06) ^ƒ^

TIMI: thrombolysis in myocardial infarction, according to the TIMI Study Group (1985); MBG: myocardial blush grade, according to the Zwolle Myocardial Infarction Study Group (VAN’T HOF AW ,1998); PPCI: primary percutaneous coronary intervention; ECG: electrocardiogram; STR: ST elevation resolution; No STR: number of ST Elevation resolution

ƒ comparison of TIMI 0 and 1 versus, TIMI 2 and 3, MBG 0 and 1 versus MBG 2 and 3, and patients with and without STR at 90min and at 24hours by Fisher Exact Test;

Ţ: p-value calculated by Student t-test (95%IC,0.32-31.28);

¶ tirofiban percentage;

Ω placebo percentage.

No variable, including treatment with tirofiban or stent post-dilation during PPCI, had predictable value for the occurrence of NR.

## Discussion

Despite the small sample population of the study, randomization assured homogeneity between the two experimental groups for all the baseline characteristics such as age, gender, obesity or overweight, coronary artery disease (CAD) risk factors, previous use of anti-ischemic and anti-atherosclerotic medications. Also, time elapsed between symptom onset and presentation to the cath lab, degree of LV dysfunction and other characteristics that may influence the PPCI results (e.g. CAD severity and extension and duration of procedures) were comparable between the groups . Only depression of left ventricular ejection fraction was more marked in the group treated with placebo, but this may reflect a worse baseline condition or a result of a less successful PPCI.

As expected, the pre-PPCI epicardial and myocardial flow grades, expressed as TIMI-0 and MBG-0, were markedly predominant in both groups. In a small number of cases we observed that spontaneous recanalization of the culprit artery had already begun when the diagnostic angiography was performed.

Taking into consideration the angiographic criteria, NR was diagnosed in 32.8% of patients, 25% of the tirofiban group and 38.3% of the placebo group (TIMI flow grade < 3). This is a higher incidence of NR than has been predicted in the literature.^5,6^ In contrast, by the angiographic criteria of myocardial reperfusion (MBG < 2), NR was detected in only 7.8% of patients (all from the placebo group), an incidence similar to that referred in previous investigations.^10,11^ Although these differences between the groups did not attain statistical significance, they show a tendency for a better result of PPCI in the group treated with tirofiban, which contrasts with previous findings.^12^ Also, these results confirm that angiographic analyses can detect only part of the occurrence of NR. When the electrocardiographic criteria for NR (STR < 70%) was used, the first analysis at 90 minutes led to the diagnosis of NR in 29 (50%) patients, 10 (41.6%) in tirofiban group and 19 (55.8%) in placebo group; and the second analysis at 24 hours diagnosed NR in 26 patients (45%), 7 (29.2%) in tirofiban group and 19 (56%) in placebo group. The differences between the groups were not statistically significant, but there was a trend toward a benefit for a reduction in NR incidence in the tirofiban group (p=0.06). When the severity of NR was assessed taking into account a STR < 30%, severe NR was not detected in the tirofiban group at 90 min, but it occurred in 9 (26.5%) patients of the placebo group at 90 minutes (p=0.01). Also, at 24hours, severe NR was present in 1 (4.2%) tirofiban patient and in 8 (23.5%) placebo patients (p=0.06). These findings also support the notion of a possible beneficial reduction of NR when tirofiban is administered early during the PPCI for STEMI patients within the 12 hours after the onset of symptoms.

In addition, the differences in NR incidence between the groups were not related to technical aspects of the PPCI procedure, such as characteristics of devices (balloons, stents and others), or strategy and duration of therapy,^12,13^ that may affect the occurrence of NR due to distal embolization, since these parameters were not different between the groups. Also, adjunctive therapy (nitrates, adenosine) was used in similar proportions in the two groups. The use of manual thrombus aspiration device was more frequent in the placebo group, but since it was not a protocol-mandated procedure, this may only indicate that this was performed at the discretion of the operator (who was blinded to the infusion solution).

Our sample calculation was mainly based on a previous clinical trial and a meta-analysis, reporting that only 35% of the STEMI patients treated with PPCI would achieve an optimal epicardial and myocardial perfusion, i.e., TIMI grade 3, MBG grade 2 or 3 and STR > 70%, when the angiographic and electrocardiographic criteria are taken into consideration altogether . Therefore, NR would be present in 65% of the patients.^7,14,15^ Our findings support the need of a more integrated and complementary use of all diagnostic criteria for NR. In fact, the differences of STR between the analyses at 90min and 24h show that NR exhibits a dynamic time behavior after PPCI, with a possible worsening of the condition at a later stage, as observed with one patient in the tirofiban group.

The lower overall incidence of NR in our sample population in comparison with those studies on which our hypothesis was based may be due to the fact that oral dual antiplatelet therapy was not yet in widespread clinical practice in those studies. In fact, our results are consistent with the findings of other studies that showed that oral double antiplatelet therapy as a routine pre-PPCI management was superior to only ASA to prevent hard clinical endpoints in STEMI patients.^16,17^

The results of the current study are also in line with those of a North American registry on more than 300,000 patients with STEMI treated with PPCI, reporting a NR frequency of only 2.3% according to the angiographic TIMI grade criteria. Of note, GPI was used in more than 70% of these patients.^18^ However , studies with abciximab that showed a benefit in mortality and reinfarction compared to placebo were performed when dual oral antiplatelet therapy had not yet been adopted in routine,^19,20^ and more recent studies with low molecular weight GPI reported similar results.^12^

The variability of the incidence and severity of NR reported in several studies may also be due to the use of only one of the two existing angiographic criteria. Also, there are disagreements between angiographic and electrocardiographic criteria, as we found in the present study.^11^ For the assessment of myocardial reperfusion after PPCI, inadequate STR should be considered a more reliable criterion for NR than the angiographic one , because it carries inherent prognostic information.^21^ Although in most of studies assessing NR with the STR criteria, only one analysis was performed after 1hour of treatment,^22,23^ we also analyzed STR at 24hours, considering that NR is a dynamic phenomenon in a sizeable proportion of patients. It is important to emphasize that these ECG analyses can be performed almost without additional cost, in contrast with the use of magnetic resonance, myocardial scintigraphy and doppler echocardiography , to assess myocardial reperfusion in the infarct area.

In conclusion, the results of this pilot study suggest that the in-lab upfront use of tirofiban in STEMI patients treated with PPCI may reduce the occurrence and severity of NR, and provide support for a definitive trial to assess the role of GPIs to prevent NR and reduce hard clinical endpoints in this context. The role of IIb/IIIa inhibitors also remains to be determined for patients being treated with novel antagonists of the P2Y12 receptor, such as prasugrel and ticagrelor, possibly in boluses administration to reduce the bleeding risk and to reduce the thrombotic risk during the initial gap in the effectiveness of the antiplatelet regimen. The recently approved inhibitor cangrelor may also constitute a valuable option in the context, due to the inherent fast onset and clearance of its antiplatelet effect.^24^

### Limitations

We acknowledge that the study has several limitations, such as a small sample size. Because of that, even with unrestricted randomization, one group (placebo) was larger than the other (intervention) group. In addition, it was left at the discretion of the operator to use resources such as manual thrombus aspiration device, which was used in 11 patients. Second, we did not measure serum cardiac enzymes to assess the occurrence of myocardial necrosis. Third, although drug eluting stents may now be considered the first choice in PPCI, only bare metal stents were available in our institutions, and we do not think that this would affect our short-term results. Finally, we did not assess patients' clinical data after hospital discharge, since their follow-up since then was performed by their referring physicians.
